# CFTR Modulator Response in Nasal Organoids Derived from People with Cystic Fibrosis

**DOI:** 10.3390/cells14231914

**Published:** 2025-12-02

**Authors:** Stefania Lo Cicero, Germana Castelli, Aurora Ceci, Anna Maria Cerio, Giovanna Blaconà, Mariarita Virgulti, Sara Allushi, Giovanni Sette, Francesca Spadaro, Felice Amato, Paola Melotti, Claudio Sorio, Giuseppe Cimino, Mauro Biffoni, Marco Lucarelli, Adriana Eramo

**Affiliations:** 1 Department of Oncology and Molecular Medicine, Istituto Superiore di Sanità, 00161 Rome, Italy; stefania.locicero@iss.it (S.L.C.); germana.castelli@iss.it (G.C.); aurora.ceci@iss.it (A.C.); annamaria.cerio@iss.it (A.M.C.); giovanni.sette@iss.it (G.S.); mauro.biffoni@iss.it (M.B.); 2 Department of Experimental Medicine, Sapienza University of Rome, 00161 Rome, Italy; giovanna.blacona@uniroma1.it (G.B.); mariarita.virgulti@uniroma1.it (M.V.); sara.allushi@uniroma1.it (S.A.); marco.lucarelli@uniroma1.it (M.L.); 3 Confocal Microscopy Unit, Core Facilities, Istituto Superiore di Sanità, 00161 Rome, Italy; francesca.spadaro@iss.it; 4 Department of Molecular Medicine and Medical Biotechnologies, University of Naples Federico II, 80131 Naples, Italy; felice.amato@unina.it; 5 CEINGE Biotecnologie Avanzate Franco Salvatore S.c.a.r.l., 80145 Naples, Italy; 6 Cystic Fibrosis Centre, Azienda Ospedaliera Universitaria Integrata Verona, 37126 Verona, Italy; paola.melotti@aovr.veneto.it; 7 Clinical Genetics Unit, Azienda Ospedaliera Universitaria Integrata Verona, 37126 Verona, Italy; 8 Department of Medicine, Division of General Pathology, University of Verona, 37134 Verona, Italy; claudio.sorio@univr.it; 9 Cystic Fibrosis Reference Center of Lazio Region, AOU Policlinico Umberto I, 00161 Rome, Italy; gi.cimino@policlinicoumberto1.it; 10 Pasteur Institute, Cenci Bolognetti Foundation, Sapienza University of Rome, 00161 Rome, Italy

**Keywords:** airway stem cells, airway organoids, disease modeling, cystic fibrosis, *CFTR* pathogenic variants, targeted therapy

## Abstract

Despite the progressive extension of *CFTR* variant eligibility to the triple combination of elexacaftor/tezacaftor/ivacaftor (ETI), most rare *CFTR* pathogenic variants remain ineligible for CFTR modulators. It is crucial to determine whether unexplored variants are rescuable by clinical modulators and to identify innovative therapeutic strategies for rescuing non-responder variants. The approach known as “theratyping” (in vitro testing of genotypes) has been accepted by the Food and Drug Administration (FDA) for the extension of clinical modulators’ approval for in vitro responding genotypes. We used one of the most advanced models for theratyping: organoids derived from nasal epithelia of people with cystic fibrosis (pwCF). We optimized the forskolin-induced swelling (FIS) of organoids to assess CFTR basal or modulator-restored function. Nasal organoids mimicked the original epithelial tissue, CFTR residual activity, and modulator response. We set up the FIS assay using nasal organoids with reference genotypes and theratyped 38 rare (non-F508del) *CFTR* genotypes, either eligible or non-eligible for FDA approval, for treatment with ETI or ivacaftor. We found strong correspondence between the in vitro response of *CFTR* variants to modulators and their FDA approval status. Additionally, some previously uncharacterized *CFTR* variants have proven responsive to clinical modulators, with significant therapeutic implications. These results suggest that the nasal organoid FIS assay, pending confirmation of the prediction in the corresponding pwCF, might be considered as a powerful in vitro tool to predict modulator efficacy in each pwCF, guiding out-of-label prescription in CF, and to identify uncharacterized variants responsive to modulators. This approach may allow comparison of the efficacy of different therapeutics or the identification of innovative strategies for non-responding genotypes, improving personalized therapy and quality of life for pwCF.

## 1. Introduction

Cystic Fibrosis (CF) is the most common rare genetic disease in the Caucasian population (1/3000 newborns) [1,2]. It is an autosomal recessive genetic disorder caused by disease-causing variants of the CF transmembrane conductance regulator (*CFTR*) gene [3,4]. CFTR is expressed in epithelial cells of many organs, including the lung, intestine, and pancreas, where it regulates ion transport, salt balance, pH, and mucus viscosity [1,2,5]. More than 2100 *CFTR* gene variants have been identified, opening the way for personalized therapy for people with CF (pwCF); however, this is currently restricted to the most frequent *CFTR* pathogenic variants [6,7,8]. The CFTR modulator drug ivacaftor (Kalydeco) has been approved to potentiate gating pathogenic variants, and the extremely effective triple combination of correctors elexacaftor/tezacaftor and potentiator ivacaftor (ETI) has been approved in recent years for genotypes bearing at least one copy of the F508del *CFTR* variant [7,9,10,11]. This triple combination is registered as Trikafta under the U.S. Food and Drug Administration (FDA) or Kaftrio under the European Medicines Agency (EMA). It has been subsequently approved by the FDA for 177 (in 2021) and additional 94 (in 2024) rare *CFTR* variants (https://www.accessdata.fda.gov/drugsatfda_docs/label/2024/212273s013lbl.pdf, accessed on 2 October 2025) [12]. The drug indication extension was based on in vitro pharmacological tests, which utilized in vitro test methods using Fischer Rat Thyroid (FRT) cells overexpressing specific *CFTR* variants and electrophysiological assays (Ussing chamber assay) [13]. Clinical studies, in particular, two French Compassionate programs recruiting pwCF carrying a large number of rare *CFTR* pathogenic genotypes not including the F508del variant (non-F508del), showed correspondence between the clinical efficacy of the triple combination and the response of the corresponding pwCF-derived cells studied in vitro (electrophysiological response) [14,15]. The in vitro testing of *CFTR* rare variants for the prediction of modulator response (theratyping) has increased relevance for personalized therapy in European populations, in which the frequency of rare *CFTR* variants is higher compared to US populations and higher in southern versus northern Europe, reaching up to 30% of pwCF [16].

FRT cell models have been largely used for the in vitro testing of modulator efficacy [17]. However, although they provide highly specific experimental settings and clear results, they may not represent a highly suitable tool for all kinds of *CFTR* variants. Innovative approaches have been introduced for theratyping, based on human primary intestinal or respiratory cells, that provide personalized models that more faithfully reproduce pwCF tissue. Nevertheless, significant obstacles have been encountered in the expansion of primary cell cultures, resulting in insufficient material for experimentation in the past [18,19,20]. Recently, primary nasal cells have been used to demonstrate that in vitro models can predict clinical modulator activity, also utilizing electrophysiological assays in Ussing chambers, and have proven to be capable of identifying pharmacological responses that may not be detected with previously used cell line models [21,22]. Among the most efficient in vitro approaches used to expand primary airway cells is the conditionally reprogrammed cell (CRC) approach, which we have optimized in recent years [23,24,25,26,27,28,29,30]. This approach enables the isolation of nasal epithelial primary cultures, with 100% efficiency and the acquisition of large amounts of cells for experimentation, through the conditional reprogramming and proliferation of epithelial respiratory stem cells [23,24,25,31]. These features also allow the generation of differentiated epithelia resembling the original tissue, in the absence of transformation (as in cell lines), or genetic manipulation (as in FRT cell models). Both 2D respiratory cell tissues (the Air Liquid Interface, ALI-cultures) and 3D nasal organoid models can be obtained. They replicate the original tissue proving highly suitable for in vitro specific assays aimed at determining CFTR basal and modulator-restored activity (theratyping) [19,23,25].

Here, we used pwCF-derived nasal organoids obtained through the CRC approach from 38 different pwCF with *CFTR* genotypes eligible (FDA+) or non-eligible (FDA−) for FDA-approved modulators and tested their basal/residual or modulator-restored CFTR activity in vitro. The response of each genotype was evaluated based on the forskolin-induced swelling (FIS) of organoids with the aim of assessing possible correlations between the in vitro response to ETI and the clinical approval status for ETI, used as a surrogate for clinical efficacy. Control organoids bearing wt *CFTR* (N/N) or healthy carriers of *CFTR* pathogenic variants (N/m) were used to define the parameters of functional CFTR activity. The clinically approved/responsive F508del/F508del homozygous genotypes, genotypes with a single copy of the approved variant (F508del/null) and null/null genotypes, were used for the pharmacological validation of models, to define the parameters of clinically relevant in vitro responses to CFTR modulators. Null is a *CFTR* variant with no residual function and unresponsive to modulators, specifically a nonsense variant.

We found correspondence between the FDA approval of CFTR modulators for the genotypes and the in vitro responsiveness of organoids through FIS assay. Notably, some (uncharacterized) variants proved to be responsive in vitro, paving the way for their future eligibility in modulator therapy. This approach may be valuable for pwCF, who may benefit from modulator therapy, when a formal demonstration of activity cannot be provided by clinical trials.

## 2. Materials and Methods

### 2.1. Nasal Brushing Processing and CRC Culture

Human nasal epithelial samples were collected in accordance with consent procedures approved by the National ethics committee for clinical trials of public research bodies (EPR) and other national public institutions (CEN) at the Istituto Superiore di Sanità (ISS), (Prot. PRE BIO CE N. 0055217 29.11.2023) and by the Institutional Ethics Committee in Verona (ref. 3397CESC prot.CRCFC-CFTR050 approval #1345 on 16 December 2021). Each individual signed an informed consent form, confirming their participation in the study (they consented to the collection of samples, the publishing of data, and publication of the results). Nasal epithelial cells were obtained from both nostrils via the cytology brushing (Doctor Brush, AIESI, Naples, Italy) of inferior turbinates; each sample was collected and placed in a single 15 mL conical tube filled with DMEM (Gibco, Waltham, MA, USA, code 31765-027) and antibiotics (Penicillin/Streptomycin and Amphotericin B). Samples were repeatedly washed, and recovered cells were cultured according to the conditionally reprogrammed cell (CRC) methodology that we previously published [23,24,25]. Briefly, epithelial cells were co-cultivated with irradiated (30 Gy) murine J2 Swiss 3T3 fibroblasts (Kerafast, Boston, MA, USA, code EF3003) in F medium 3:1 *v*/*v* F-12 Nutrient Mixture Ham (Gibco, Carlsbad, CA, USA, code 11765054): DMEM (Gibco, code 31765-027) supplemented with 5% Fetal Bovine Serum (Euroclone, Milan, Italy, code ECS01180L); 0.4 μg/mL of hydrocortisone (Sigma, St. Louis, MO, USA, code H0888); 5 μg/mL of insulin (Sigma, code 91077C); 24 μg/mL of adenine (Sigma, code A2785); 8.4 ng/mL of cholera toxin (Sigma, code C8052) in the presence of 10 μM Rock inhibitor Y-27632 (Selleck, Munich, Germany, code S1049); and 10 ng/mL of EGF (Peprotech, Cranbury, NJ, USA, code AF100-15). Fibroblasts were cultured in DMEM supplemented with 10% characterized MSC-qualified USDA-approved Fetal Bovine Serum (HyClone™, Gibco, Carlsbad, CA, USA, code 12662029) and irradiated when 80% confluence was reached. Gamma irradiations from a 137Cs source were carried out at the Istituto Superiore di Sanità (ISS, Rome, Italy) using the Gammacell Exactor 40 (Nordion, Ottawa, Canada). All cells were maintained at 37 °C in a humidified incubator with 5% CO_2_.

### 2.2. CRC-Derived Organoid Generation and Forskolin-Induced Swelling (FIS) Assay

CRCs were suspended at 1.1 × 104 cells/50 µL in growth factor-reduced Matrigel (Corning 354230) using careful pipetting to generate a single-cell suspension, avoiding the generation of air bubbles. This mixture was seeded into 24-well plates, creating a flat “drop” of Matrigel. The plates were incubated at 37 °C and 5% CO_2_ for 30 min to allow the Matrigel to set. PneumaCult Airway Organoid Seeding Medium (0.5 mL) was added to the wells to cover the Matrigel drop. Medium was replaced with fresh medium after 4 days; after a total of 7 days, cells were shifted to PneumaCult™ Airway Organoid Differentiation Medium (PneumaCult™ Airway Organoid Kit # 05060, Stem Cell Technologies, Cambridge, UK, 05001), and the medium was replaced every other day for an additional 4 weeks. Mature organoid 3D structures formed in the presence of a lumen and a slightly thickened spheroid wall, indicating a pseudostratified epithelium with motile cilia visible under an optical microscope (high-magnification objective). For the FIS assay, organoids were pretreated with the corrector combination of tezacaftor (10 µM) plus elexacaftor (3 µM) for 48 h. Then, spheroid images were captured (10× magnification) at 48 h after corrector treatment (time 0), using a time-lapse imaging station (Olympus, Tokyo, Japan). Each organoid was monitored, and after 2 days of subsequent stimulation with the same correctors, plus 5 µM of Ivacaftor VX770 (Selleck Chemicals, Houston, TX, USA, code S1144) and 10µM of forskolin (Selleck Chemicals, code S2449), new images of the same organoids were taken (time 1 in figures) to monitor and assess spheroid swelling. Three independent experiments were performed, and N = 10 spheroids in each well per each experimental condition were analyzed for each experiment (triplicate). The area of each spheroid was analyzed by manually delineating the outer surface using ImageJ software, version 4.78.2 before and after the forskolin treatment to avoid potential biases due to organoid size heterogeneity. Spheroid outer area measurements (basal and after stimulation) were imported into Microsoft Excel, and the fold change was calculated for each individual spheroid with respect to the same untreated ones.

### 2.3. Immunofluorescence and Confocal Laser Scanning Microscopy (CLSM) Analysis of Organoids

For CLSM analysis, CRC-derived nasal organoids were generated in 8-well chamber slides (Corning 354108) and differentiation was induced by culture in Pneumacult Airway Organoid medium for 4 weeks, as was conducted for the FIS assay. Intact organoids in Matrigel were fixed with 2% paraformaldehyde for 30 min at room temperature, and permeabilized with 0.5% Triton-X-100 for 15 min at room temperature. Primary mouse anti-acetylated α-tubulin (Sigma) or anti-CFTR (Ab570, obtained via the Cystic Fibrosis Foundation) combined with rabbit anti-Mucin 5B (HPA008246, Sigma) or anti-cytokeratin 5 (PA5-32465, Invitrogen, Carlsbad, CA, USA) antibodies were added in 0.5% Triton-X-100/3% BSA/3% FCS and incubated for 90 min at room temperature, followed by incubation with Alexa Fluor-488 F(ab)2 fragments of goat anti-mouse IgG and Alexa Fluor-594 F(ab)2 fragments of goat anti-rabbit IgG (Thermo Fisher Scientific, Waltham, MA, USA) for 1 h at room temperature. Nuclei were stained with 4′,6′-diamidino-2phenykindole (DAPI, Thermo Fisher Scientific). Coverslips were finally mounted with Vectashield mounting medium for fluorescence (Vector Laboratories, Newark, CA, USA), after removal of the chamber apparatus. CLSM observations were performed with a Zeiss LSM980 microscope (Zeiss, Oberkochen, Germany), using a 40×/1.40 NA oil objective and excitation spectral laser lines at 405, 488, and 594 nm. Image acquisition and processing were carried out using the Zeiss confocal software Zen 3.3 (Blue edition) and Adobe Photoshop CS5 version 12.0.0.0 software program (Adobe Systems). Signals from different fluorescent probes were taken in sequential scan settings. Several organoids for each labeling condition were analyzed, and representative results are shown.

### 2.4. Immunoblot for Validation of Organoids Differentiation

After the FIS assay, the organoids were recovered for immunoblotting assays. The medium was removed, and organoids in Matrigel were washed with PBS. The Matrigel matrix was disrupted with vigorous but careful pipetting with cold PBS, and a suspension of organoids was generated. The suspension recovered was centrifuged twice at 5000 rpm for 5 min in a minicentrifuge, and the pellet (derived from 3 wells for each condition) was lysed in 45 µL of lysis buffer (1% NP40, 0.15 mM NaCl, and 50mM TRIS-HCl pH 7.4 with protease and phosphatase inhibitors). Total protein lysate (20 μg) of from each sample was resolved on 3–8% polyacrylamide gel electrophoresis NuPAGE Tris–Acetate (Invitrogen, Carlsbad, CA, USA, EA03752BOX) and transferred to nitrocellulose membranes (GGE Healthcare Life Science, Marlborough, MA, USA, AmershamTM ProtranTM, 10600018). The primary antibodies used were mouse monoclonal CFTR-596 (CFTR Antibody Distribution Program Cystic Fibrosis Foundation, UNC-Chapel Hill, dilution 1:5000), mouse anti-acetylated α-tubulin (Sigma clone 6-11B-1, #T6793), anti-Keratin 14 antibody (Biolegend, San Diego, CA, USA, cat. 906004), and mouse monoclonal anti-nucleolin (C23, MS-3 Sant Cruz, dilution 1:500). The peroxidase-conjugated secondary antibodies used were from Amersham™ (NA931V, NA934V, dilution 1:4000). ECL images were acquired using Image lab software (latest v. 6.1., Chemidoc XRS+, Biorad, Hercules, CA, USA) in a linear range of exposure.

### 2.5. CFTR Mutational Analysis

Genomic DNA was extracted from the CRCs using the QIAamp DNA Blood midi kit (Qiagen, Hilden, Germany 51183), and fluorimetric quantification was performed (Qubit, Invitrogen, CA, USA). Proximal 5′-flanking, all exons and adjacent intronic zones, and the 3′-UTR of the *CFTR* gene (RefSeq NM_000492.4, NG_016465.4) were sequenced using the Sanger cycle sequencing protocol (ThermoFisher Scientific, Waltham, MA, USA), as previously described, and a genetic analyzer (ABI PRISM 3130xl; Applied Biosystems, Foster City, CA, USA) [32]. Genotype analysis was completed usinglmultiplex ligation-dependent probe amplification (SALSA MLPA probemix P091 *CFTR*, MRC Holland, Amsterdam, The Netherlands, EKI-FAM, P091-100).

## 3. Results

### 3.1. Patients’ Characteristics

Thirty-eight pwCF carrying rare (non-F508del) *CFTR* variants were selected for this study, among those available at the Cystic Fibrosis Reference Center of Lazio Region, AOU Policlinico Umberto I (Rome, Italy), and at the Cystic Fibrosis Center at AOUI of Verona, to test their in vitro responses to modulators. Three non-CF subjects with wild-type *CFTR* (N/N) and three healthy carriers of *CFTR* pathogenic variants (N/m) were selected as references for the wild-type allele in double or single copies, respectively, with both genotypes producing an amount of non-mutated CFTR sufficient for function. Six F508del/F508del genotypes and three genotypes carrying a single F508del copy with a *CFTR* variant in trans with an allele with no residual function and unresponsive to modulators (F508del/null)—both genotypes eligible and clinical responders to ETI—were selected as references for the clinically relevant in vitro response to modulators (responsive allele in double or single copy, respectively).

PwCF data are reported in Table S1, including genotypes and parameters describing their clinical status.

### 3.2. Generation and Phenotypical, Functional, and Pharmacological Validation of Nasal Organoids

Nasal brushing cells collected from pwCF were cultured under the conditionally reprogrammed cell (CRC) approach, determining the prominent expansion of respiratory basal stem cells, following our previously published protocols [19,23,24,25,31,32]. Both CRCs and CRC-derived organoids were fully characterized, as described in the Supplementary Materials and in Figure S1.

The forskolin-induced swelling of organoids (FIS) assay was first performed for the reference genotypes to determine the parameters of organoid swelling induced by functional CFTR (in wt-*CFTR* or healthy carrier organoids), as well as the extent of CFTR rescue achievable through ETI treatment in the clinical responder genotypes with two (F508del/F508del) or one (F508del/null) copies of responder allele F508del. Finally, the W1282X/W1282X genotype (null/null) was used as a standard for the non-responding genotypes [25]. Reference genotype samples and their FIS assay responses are reported in Table S2, and the results are reported in Figure S2. The FIS for non-CF and carrier organoids in the absence of modulators was 2.32 ± 0.26 and 2.22 ± 0.21, respectively, which is in line with the healthy phenotype of carrier individuals. Organoids carrying the F508del allele, in double (F508del/F508del) or single (F508del/null) copies, were treated with fsk, fsk plus the potentiator ivacaftor, or fsk plus the triple combination of ETI. Both genotypes did not respond to fsk or fsk+ivacaftor, but showed a marked fsk+ETI response, as expected for clinical responders to ETI, lacking residual function (no response to fsk), and not bearing a pure gating defect (no response to ivacaftor). For F508del/F508del, the mean FIS_ETI_ was 1.94 ± 0.19; for the genotype carrying a single F508del allele (F508del/null), the mean FIS_ETI_ was 1.66 ± 0.15 (increase with respect to non-swelling organoids). In contrast, W1282X/W1282X organoids did not undergo swelling after fsk+ETI treatment, as expected, showing a FIS_ETI_ value of 1.02 ± 0.02. FIS values for the different reference genotypes tested are reported as histograms in Figure S2A, and representative images of FIS assay organoids for wild type (wt), carrier, F508del/F508del, F508del/null, and W1282X/W1282X organoids are reported in Figure S2B. Here, we showed the cumulative CFTR activity (residual function + the modulator-induced rescue of abnormal CFTR function) through comparison with the FIS_ETI_ of non-swelling organoids (nontreated, NT), which would be consistent with the overall activity of CFTR in modulator-treated patients. Overall CFTR activity, in turn, would result in sufficient or insufficient ion exchange and hydration in the epithelial tissue. Thus, this analysis, conducted on clinical responder genotypes, provided the extent of CFTR activity in vitro that could be considered sufficient for patient benefit.

On the other hand, it is also relevant to clarify, and better quantify, the specific effect of modulators in rescuing defective CFTR over the residual function. To this aim, we calculated the swelling changes induced by fsk+ETI, compared to control organoids treated with fsk. This analysis provides the extent of ETI-induced increase in CFTR activity over the basal residual CFTR activity. These different analytical approaches provided similar results (Figure S2A,B), as expected based on the limited residual function of the F508del genotypes.

### 3.3. ETI Response of 38 Rare CFTR Genotypes in the FIS Assay of Nasal Organoids Correlated with FDA Approval Status for ETI

FIS assays were next performed for 38 rare *CFTR* pathogenic genotypes (not including the F508del variant) that were either FDA+ or FDA−. All samples were examined before the experiments to verify the *CFTR* sequence, organoid differentiation, and cilia movement by microscope observation.

**Figure 1:**
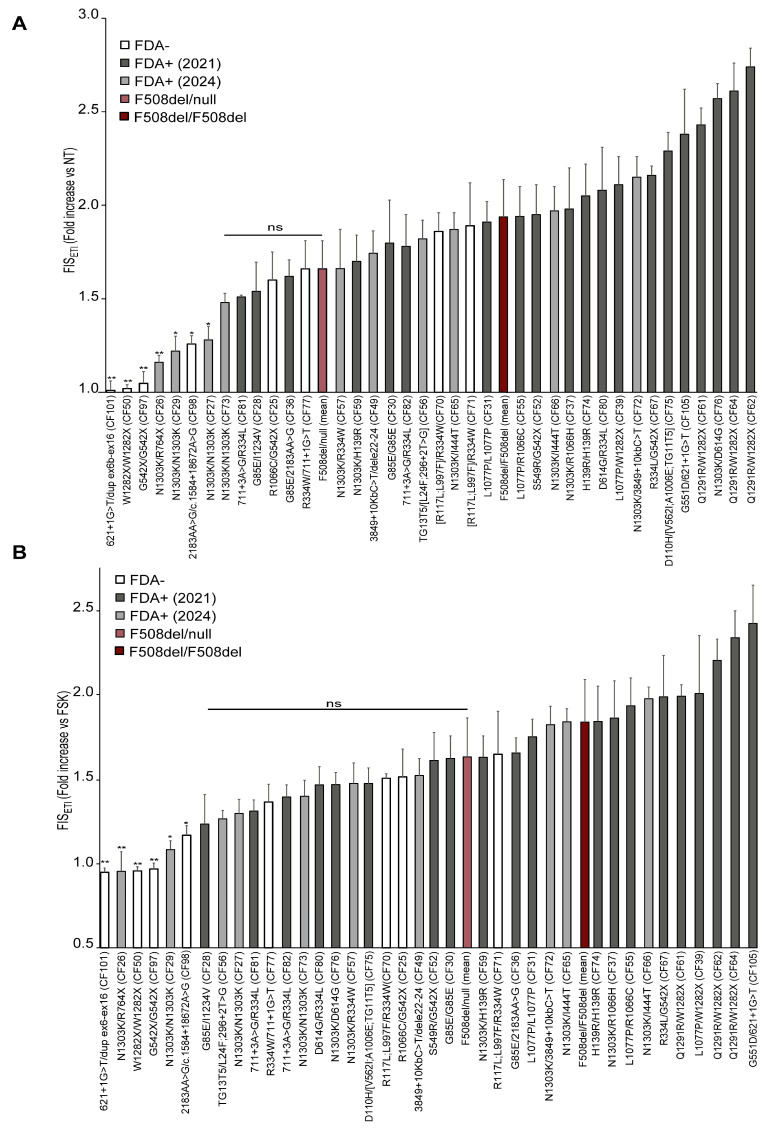
General correspondence of in vitro response of nasal organoid FIS assay to ETI and FDA approval status for Trikafta. Nasal organoid FIS_ETI_ assay results of 38 CF rare (non-F508del) genotype samples, and 2 control genotypes (1 copy and 2 copies of F508del). Samples are distributed in the graph based on increasing FIS_ETI_ values. Mean FIS_ETI_ responses of reference genotypes F508del/F508del and F508del/null are also included in the graph to visualize the threshold values of clinical responders (specifically, those of the F508del/null genotypes were considered for the lower threshold limit associated with clinical response). Organoids were pretreated with 10 µM tezacaftor and 3 µM elexacaftor (ET) for 48 h and then treated with the same correctors and 5 µM ivacaftor in combination (ETI) plus 10 µM forskolin, for an additional 48 h. The *y*-axis indicates organoid swelling values, each normalized to the untreated sample baseline (**A**) or to the fsk-treated organoids (**B**). Values are mean of three independent experiments. White bars indicate Trikafta non-FDA-approved genotypes by FDA; dark gray bars are genotypes approved for Trikafta in 2020; the light gray bars show genotypes approved for Trikafta in 2024; dark red bar represents FIS_ETI_ value of F508del/F508del genotype (mean of 6 different samples); and the light red bar represents FIS_ETI_ value F508del/null genotypes (mean of 3 different samples). For samples on the left of the F508del/null genotypes, the statistical significance of the difference in their ETI values versus those of the F508del/null genotypes was analyzed via ANOVA: *p* < 0.05 *, *p* < 0.01 **.

Their basal/residual (FIS) or ETI-restored (FIS_ETI_) CFTR activity in vitro was evaluated in the FIS assay; the results are listed in Table 1 and the corresponding FIS_ETI_ histograms are reported in Figure 1.

FDA+ genotypes exhibited higher FIS_ETI_ values, generally, and higher than the F508del/null group of genotypes, while most FDA− genotypes were distributed at lower values and a clear correspondence between clinical approval and FIS response was found.

*CFTR* genotypes including FDA+ (years 2020 and 2024) variants generally displayed a marked response to ETI, including those carrying the G85E, L1077P, R1066H, H139R, R334L, D614G, R117L, G551D, D110H, TG13T5, 3849+10Kb C>T, Q1291R, 711+3A>G, and S549R variants, which is in line with their eligibility for ETI, and as expected, based on previous reports. Also, R117L and L997F as well as V562I and A1006E should be included in this list, although they were found in complex alleles in our case series and are considered FDA+ as single variants.

In contrast, the following severe FDA− variants were confirmed to be non-rescuable by ETI in the FIS assay (Figure 1, first genotypes on the very left side of the graph): W1282X, 621+1G>T, dup ex6-ex16, R764X, G542X, 2183AA>G, and c.1584+18672A>G.

Importantly, some rare genotypes, still not eligible for treatment with ETI, as R1066C/G542X (CF25) and R334W/711+1G>T (CF77), displayed FIS_ETI_ values comparable to those of the F508del/null clinical responder (FIS_ETI_ values were slightly lower than F508del mean FIS value, but these differences were not statistically significant). Thus, these genotypes could be considered in the range of clinical responders to ETI, with important implications for the corresponding pwCF or other pwCF with the same genotypes.

Some genotypes containing FDA− complex alleles displayed ETI responses, including TG13T5/[L24F;296+2T>G] (CF56), D110H/[TG11T5;V562I;A1006E] (CF75), and [R117L;L997F]/R334W (CF70 and CF71). However, it cannot be ascertained whether the ETI responses in these three genotypes are due to the rescue of the complex allele, the single variant present on the other allele, or both. In fact, ETI is approved for at least two of these variants which are present on the second allele (TG13T5 and D110H).

Regarding the N1303K variant, we theratyped three samples carrying the N1303K/N1303K genotype (CF27, CF29 and CF73) derived from different pwCF. One of them (CF73) displayed FIS_ETI_ values comparable to those of clinical responders, while two samples (CF27 and CF29) showed a weaker response. This is in line with the general variability of this genotype, which has been reported as a genotype featuring a borderline clinical response [17,22]. A very low response was also observed in the N1303K/R764X genotype (CF26) with FIS_ETI_ = 1.16 (s.d. ± 0.04).

In taking into consideration these variable, low-to-moderate responses to ETI found for the N1303K variant in homozygous genotypes (CF27, CF29, CF73) as well as in the N1303K/R764X genotype (CF26), it is likely that the N1303K variant might have a minor contribution in the ETI response observed in the N1303K/I444T (CF65 and CF66), N1303K/3849+10kb C>T (CF72), N1303K/D614G (CF76), N1303K/R1066H (CF37), N1303K/H139R (CF59), and N1303K/R334W (CF57) genotypes. Thus, the remarkable ETI-induced CFTR rescue observed in these samples could depend mainly on the non-N1303K allele (with important clinical implication for the non-approved I444T or R334W variants).

In the R1066C/G542X genotype (CF25), we can reasonably assume that response to ETI was likely mediated by the R1066C variant, as the nonsense G542X variant has been widely reported to be non-rescuable with ETI, in line with our results proving that nasal (CF97) and intestinal organoids from the same individual carrying the G542X/G542X genotype are non-responders to ETI [33].

Similarly to the analyses performed for reference genotypes (Figure S2B), to determine the specific efficacy of ETI in rescuing abnormal *CFTR* variants, we compared the FIS values of fsk+ETI-treated organoids (FIS_ETI_) with those of FIS relative to the fsk-only treatment. Some variations between the two approaches occurred, relative to those genotypes endowed with residual function, as the residual function was counted in the first analysis (Figure 1A) and not included in the FIS_ETI_ versus fsk-treated organoid analysis (reported in Figure 1B). Thus, we found generally similar genotype responses, and correspondence with the ETI approval was maintained, except for sample CF27 (N1303K/N1303K), possibly because its FIS_ETI_ value is close to the threshold associated with clinical response.

### 3.4. Ivacaftor Response of 38 Rare CFTR Genotypes in FIS Assay of Nasal Organoids Correlated with FDA Approval for Kalydeco

The same 38 rare genotypes investigated above, including genotypes considered either FDA+ or FDA− for ivacaftor, were evaluated for ivacaftor response in the nasal organoid FIS assay, with the aim of assessing the possible correspondence of ivacaftor responsiveness in vitro with clinical eligibility. The final goal was to further validate the FIS assay/organoid model for the in vitro prediction of clinical response, also for ivacaftor, and to identify new potential ivacaftor-responsive *CFTR* variants.

**Figure 2:**
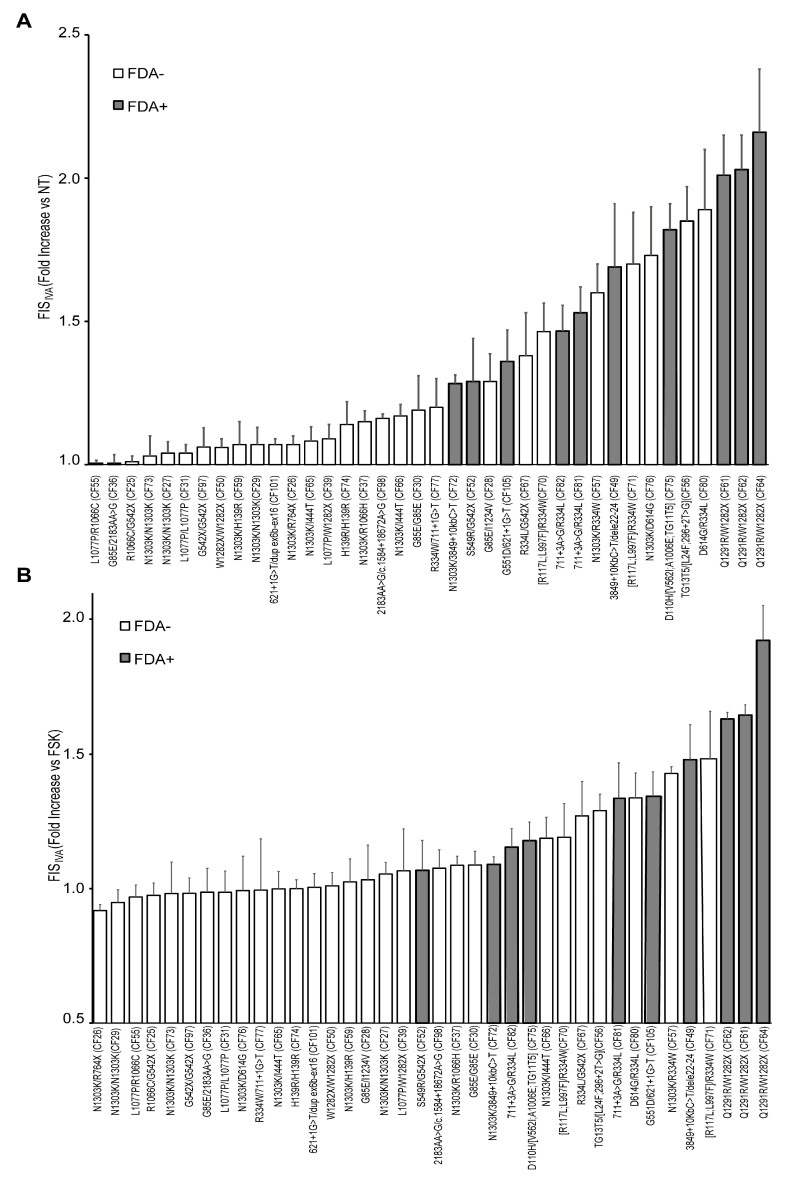
General correspondence of in vitro response of nasal organoid FIS assay to ivacaftor and FDA approval status. Nasal organoid FIS_IVA_ assay results of 38 CF rare (non-F508del) genotype samples. Organoids were treated with ivacaftor plus forskolin for 48 h, and organoid swelling was calculated like for the FIS_ETI_ assay. The *y*-axis indicates organoid swelling mean values, each normalized to the untreated sample baseline (**A**) or to fsk-treated organoids (**B**). Values are the mean of three independent experiments. Gray and white bars indicate FDA+ and FDA− genotypes, respectively.

The FIS_IVA_ values of each genotype are reported in Figure 2. All FDA+ genotypes displayed a response to ivacaftor, although at variable extents, ranging from moderate to very high values. Some genotypes displayed very high FIS_IVA_ values, comparable to the values found in non-CF organoids only stimulated with fsk. Specifically, these very high FIS_IVA_ values were observed in the genotypes bearing the Q1291R, D110H, and 3849+10kbC>T variants. In contrast, most FDA− variants displayed low or very low responses to IVA values, lower than those of the FDA+ genotypes. Thus, a solid correspondence emerged between the in vitro response to ivacaftor and the FDA approval status for Kalydeco in these studies (Figure 2). Among the FDA− genotypes that displayed promising ivacaftor response, in the range of FDA+ G551D or S549R values or higher, were G85E/I1234V (CF28), R334L/G542X (CF67), N1303K/R334W (CF57), R117L;L997F/R334W (CF70 and CF71), N1303K/D614G (CF76), TG13T5/L24F; 296+2T>G (CF56), and D614G/R334L (CF80). Considering the specific allele combination in the different genotypes, it is likely that the major contribution to ivacaftor response in these genotypes is due to the potentiation, in addition to the G551D or S549R, of the *CFTR* pathogenic variants I1234V R334L, R334W, 711+3A>G, 3849+10kb C>T, D614G, and Q1291R. These results may also suggest that other genotypes containing any of the above-listed variants may be clinically rescuable with ivacaftor.

Similarly to the analyses performed for ETI response (Figure 1B), to determine the specific efficacy of ivacaftor in rescuing defective *CFTR* variants, we compared the FIS values of fsk+IVA-treated organoids (FIS_IVA_) with those of FIS relative to the fsk-only treatment. Some variations between the two approaches occurred, relative to those genotypes endowed with residual function, as the residual function was counted in the first analysis (Figure 2A) and not included in the FIS_IVA_ versus fsk-treated organoid analysis (Figure 2B).

We observed a relative variation in results compared to the previous presentation of data (Figure 2A). This fact may depend on the higher residual function generally associated with ivacaftor-responsive genotypes, as CFTR protein may mostly have a correct conformation and it may be already located to the membrane; thus, it may retain some residual function, and its gating activity may only need to be enhanced by the potentiator.

### 3.5. Theratyping of 38 Rare CFTR Genotypes to Determine the Relative Contribution of CFTR Residual Function and of the Different Modulators to the Cumulative ETI-Induced CFTR Rescue: Selecting the Right Drug for the Right Patient

**Figure 3:**
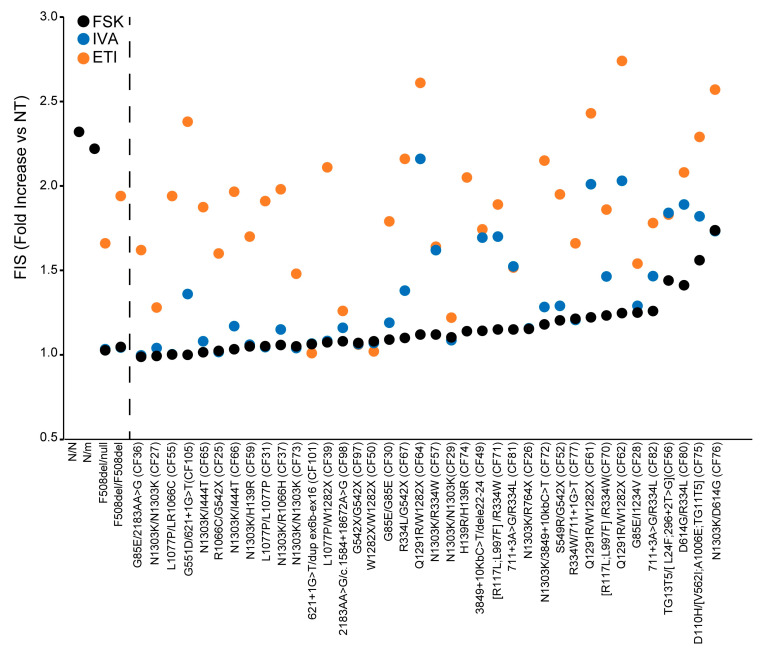
Relative contribution of CFTR residual function and of the different modulators to cumulative ETI-induced CFTR rescue. Nasal organoid FIS assay of 38 CF rare (non-F508del) genotype samples for responses to forskolin in absence of modulators (FSK, black dots), FSK plus ivacaftor (IVA, blue dots), and FSK plus the triple combination ELX/TEZ/IVA (ETI, orange dots). On the left side of the black dotted line are FIS values for control genotypes. FIS is indicated for wt (N/N) and *CFTR* carrier (N/m) genotypes. FIS, FIS_IVA_, and FIS_ETI_ are indicated for F508del in single (F508del/null) and double copies (F508del/F508del) and for rare genotypes.

We then analyzed and compared the response to ETI (FIS_ETI_), to ivacaftor (FIS_IVA_) and in the absence of modulators (FIS) of the 38 rare *CFTR* genotypes to determine the specific contribution of each drug or drug combination or of the residual CFTR function to the cumulative functional rescue obtained, following ETI treatment. These results are reported in Figure 3, where samples are distributed based on increasing values of FIS in the absence of modulators (increasing extent of residual function), and can clarify the efficacy and the requirement of each drug/drug combination to achieve a clinical-grade response. Some genotypes showed a marked FIS_IVA_ response; however, the comparison of results showed that the FIS_IVA_ was comparable to the basal FIS, indicating that the observed organoid swelling was due to the CFTR residual function rather than the potentiation of the CFTR channel. This suggests that the corresponding pwCF would not need or benefit from ivacaftor treatment, as this drug did not add further organoid swelling over that induced by fsk stimulation. In contrast, ETI administration in the same samples induced a remarkable enhancement of organoid swelling, over that observed with fsk alone or via fsk+ ivacaftor exposure (Figure 3). Other genotypes displayed a low residual function and a remarkable efficacy of ivacaftor, which in some cases was further increased with ETI, as in the Q1291R-carrying genotypes (CF61, CF62 and CF64), suggesting that the corresponding pwCF might also receive a clinical benefit from treatment with only ivacaftor, and not requiring the triple combination.

The G551D/621+1G>T (CF105) genotype was unresponsive to fsk, indicating a lack of residual function for this genotype (FIS = 1.0, s.d. ± 0.03). In contrast, as expected for the presence of the ivacaftor clinical responder variant G551D, treatment with ivacaftor induced organoid swelling, although not to a remarkable extent in this specific sample (FIS_IVA_ = 1.36, s.d ± 0.11). Interestingly, treatment with the triple combination induced significant organoid swelling, indicating that the corresponding pwCF would display a mild response to ivacaftor, while ETI would be much more effective, with important clinical implications.

Another interesting genotype is S549R/G542X (CF52), in which the response to modulators of the S549R variant can be specifically assessed, due to the presence of the unresponsive nonsense allele G542X. Fsk treatment induced a modest response in this genotype (FIS = 1.20, s.d. ± 0.04), (implying a modest CFTR residual function); ivacaftor slightly enhanced fsk-induced organoid swelling (FIS_IVA_ = 1.29, s.d. ± 0.15), which is in line with the presence of a copy of the FDA+ variant S549R. However, in this specific sample, the ivacaftor effect was not remarkable, as most of the organoid swelling was due to fsk activity (residual function) with a minor contribution by ivacaftor. In contrast, ETI administration induced a marked enhancement of organoid swelling in this genotype, exceeding the cumulative effect of residual function and potentiation with ivacaftor (FIS_ETI_ = 1.95, s.d. ± 0.16), with therapeutic implications for the corresponding pwCF.

Interestingly, the in vitro results showing modest FIS_IVA_ response for both G551D/621+1G>T and S549R/G542X samples were found to be in line with the corresponding pwCF data. In fact, these two pwCF are currently under ivacaftor treatment, but their clinical symptoms have evidenced unsatisfactory outcomes (Table S1). Thus, a request for authorization to ETI therapy is about to be submitted to the Italian regulatory agency for both patients, supported by the in vitro results described here.

## 4. Discussion

Targeted therapy with CFTR modulators has dramatically improved the clinical status and both the quality and expectancy of life for pwCF, in recent years.

After the first approval of Trikafta, in 2019, for genotypes carrying the F508del pathogenic *CFTR* variant, its eligibility has been progressively extended to 177 variants (December 2020) and lately to another 94 variants (December 2024), based on in vitro pharmacologic testing in specific cell models mainly based on the rat thyroid cell line FRT overexpressing the *CFTR* pathogenic variants of interest, and on electrophysiological assays (Ussing Chamber assay).

However, FRT overexpression models are not suitable for all classes of pathogenic variants. Examples include splicing pathogenic variants, for which a certain variable fraction of wild-type protein may be produced and which influence the residual or drug-restored CFTR activity, also on an individual patient basis; class II pathogenic variants, which are expressed at low levels due to the degradation of abnormal proteins; and nonsense pathogenic variants undergoing nonsense-mediated mRNA decay (NMD), which strongly reduce the *CFTR* mRNA and protein levels. In all these cases, CFTR variant overexpression would not faithfully replicate the endogenous CFTR levels, impacting drug response. Moreover, FRT cells, which are of rat origin, may not fully mimic human tissue or may display drug affinity different from that of a human background. Finally, FRT cell models require the use of the Ussing chamber assay, considered the gold standard for CFTR studies. However, the complexity of carrying out this assay, compared to the more achievable FIS assay, makes it less feasible. Importantly, our preliminary results, obtained from reference genotypes, showed correspondence between the Ussing chamber results and the FIS results, further substantiating the reliability of the organoid approach.

Moreover, the rarest (lacking the F508del variant) genotypes are still ineligible for modulators. Thus, therapeutic management of most of these cases, often heterozygous for different variants or carrying complex alleles, remains a difficult clinical issue.

A dramatic improvement in the in vitro testing of modulators response of rare variants (theratyping) was realized using different pwCF-derived primary cell models based on airway nasal cells, together with intestinal cell models, as these models were found to be more relevant and straightforward for pharmacological testing in vitro.

Here we focused on pwCF-derived nasal epithelial organoids obtained from nasal conditionally reprogrammed cells (CRCs) tested through the forskolin-induced swelling (FIS) of organoids. We fully characterized the model, optimized and standardized the FIS assay, and tested 38 rare *CFTR* pathogenic genotypes for their response to modulators, with particular focus on the triple combination (ETI).

The results exhibited concordance between the FDA approval status of ETI for *CFTR* genotypes and their response in vitro, as well as between the ivacaftor clinical approval status and responsiveness in vitro. This proves the suitability of the nasal organoid FIS assay for prediction of variant responsiveness to modulators, at the clinical level. The assessment of ETI response in clinically responder genotypes (F508del/null and F508del/F508del) used as reference, allowed us to estimate that the minimal threshold for an FIS_ETI_ possibly associated with clinically relevant response, may be approximately 1.5, which is the minimal value obtained for the F508del/null genotypes (Table S2). Thus, all genotypes with FIS_ETI_ values comparable (non-significantly lower) to the F508del/null FIS_ETI_ could be assumed as clinical responders. On the other hand, FIS_ETI_ scores at the threshold could also be associated with limited patient benefit from modulator treatment (suboptimal clinical response). In this regard, the mild symptomatic patients-derived organoids (CF75 and CF76) showed the basal FIS scores at this threshold, suggesting that (basal or rescued) CFTR activity at the threshold scores may be associated with mild symptoms and borderline clinical status/response. In this view, the final proof of the predictive value of the nasal organoids FIS for personalized medicine, will definitively derive from the direct comparison of in vivo/in vitro responses in the same patient/patient-derived cells. This approach also takes into account the interindividual variability in terms of disease severity and therapy responsiveness, that can be observed also in patients with identical genotypes.

Few FDA− (R1066C, R334W) *CFTR* genotypes or very recently approved for ETI (3849+10kbC>T, TG13T5) were responsive to the drug in vitro. Some FDA− genotypes (for ivacaftor) showed marked responsiveness (D614G, R334L, R334W), suggesting potential clinical efficacy of the modulators for the corresponding pwCF, with important therapeutic implications.

The CFTR residual function was investigated (response to fsk in the absence of modulators) and compared to the response to ivacaftor (plus fsk) or to the cumulative response to ETI (plus fsk) to assess the relative contribution of CFTR residual function and of the potentiator ivacaftor to the cumulative CFTR rescue obtained with the triple combination (Figure 3). This approach may provide further comprehension of the molecular/functional defects associated with the specific CFTR variants and the requirement of specific drugs/drug combinations for functional rescue. Understanding these aspects is fundamental to guiding the selection of the most appropriate drug option to achieve the best therapeutic benefit for the corresponding pwCF, which may be extrapolated to other individuals with the same genotypes. Besides the concordance of FIS response with the modulator approval status for each genotype tested, our unpublished preliminary results have shown that very few patients who started the ETI therapy showed promising clinical benefits. This is in line with in vitro findings showing clinically relevant FIS_ETI_ response on the specific patient-derived cells. As a consequence, once the concordance of the response in the FIS assay with that of the corresponding modulator-treated patient is definitively demonstrated, these studies might contribute to improving personalized therapy in the future. The organoid model could then be used to guide therapeutic choices and achieve the most satisfactory clinical efficacy, limiting both systemic toxicities associated with multiple-drug administration and costs associated with the possible use of ineffective drugs.

Of note, we found a moderate and variable response in different samples carrying genotypes containing the N1303K variant, even in different homozygous samples. This is in agreement with clinical studies reporting variable and suboptimal efficacy of ETI in pwCF carrying the N1303K variant, suggesting that further studies are needed to better understand the basis of pwCF-specific responses for this variant [17,22]. For instance, variable clinical efficacy has been achieved through ETI administration in different N1303K patients as well as in different body compartments of the same patient (lung function improvement, at least partially enhanced by lung inflammation, is more evident than sweat chloride reduction), or additionally ETI response may be possibly influenced by different tissue-specific molecular mechanisms [17,22,34,35].

Another relevant issue is that the CRC approach, allowing CF models to be obtained from each sample, may enable patient-specific in vitro theratyping, guaranteeing a more reliable prediction of the clinical response, compared to genotype-specific theratyping, taking into account the individual-specific molecular, genetic and epigenetic background, possibly influencing the therapeutic response and providing a more reliable guide for the selection of the best therapeutic option for each patient.

As a final consideration, all *CFTR* variants included in this study and in the list of 94 variants approved for ETI by the FDA in December 2024 displayed positive responses to ETI, further enforcing the value of the nasal organoid FIS assay for the prediction of clinical response and as a potential powerful tool for guiding the indication extension of modulators to additional variants.

One limitation of this study is that, for most genotypes, a single pwCF sample was studied, even though the unavailability of multiple pwCF with the same genotype can be attributed to their rarity. Indeed, the N-of-1 approach is becoming relevant in this and other fields of medicine, as has been recently discussed, and the study of these rare genotypes necessarily follows this approach [36,37]. Another approach for studying rare *CFTR* variants consists of the use of gene-modifying techniques (such as CRISPR-CAS9, or other gene editing approaches) that induce rare mutations in non-CF epithelial models [38]. This system may be relevant, although it is limited by low efficiency, possible off-target effects, and generally laborious procedures of the gene modification strategies. Importantly, the use of gene-edited non-CF epithelial models does not guarantee the patient-specific background, which is essential to replicate the levels of CFTR expression, degradation, stability, and regulation by epigenetic mechanisms, which may be different in different individuals.

Another consideration is the fact that the Committee for Medicinal Products for Human Use (CHMP) of the European Medicine Agency (EMA), has very recently approved the clinical use of the triple combination for any genotype that has at least a non-class I pathogenic variants, largely expanding the range of pwCF who can access these modulators. This was based on key data previously obtained both in vitro (using several cell models including FRT cells, primary respiratory and intestinal cells of human origin and different types of assays) and at clinical level (in the US). In fact, following the FDA approval of ETI for in vitro responding variants, as observed in theratyping studies, eligible variants were clinically treated in the US. This provided important information about the extent to which different rare genotypes respond at clinical level, which could possibly satisfy EMA requirements for extending modulator indication to a large number of *CFTR* genotypes. From this perspective, it remains fundamentally important to elucidate whether an uncharacterized pathogenic variant belongs to class I (no residual function) or whether a pathogenic variant of a different class may be non-responsive to modulators. Thus, even with a broad approval of modulator eligibility for *CFTR* variants, the in vitro nasal organoid FIS assay may be of critical importance in guiding therapeutic choices. For instance, in some genotypes, we demonstrated sufficient efficacy with potentiator ivacaftor alone, as well as the requirement of the triple-drug combination in other genotypes. Finally, the new triple combination vanzacaftor + tezacaftor + deutivacaftor (VTD, Alyftrek) was proven generally to have higher clinical efficacy compared to ETI; in this context, it would be of pivotal relevance to compare the effectiveness of ETI to that of VTD in these advanced nasal organoids models, particularly for those genotypes with unsatisfactory response to ETI [39,40]. As a final consideration, based on the reliability of the FIS assay to predict the clinical response to drugs demonstrated here for CFTR clinical modulators, CRC-based nasal organoid models may represent a powerful tool with which to test the ability of innovative experimental drugs or therapeutic approaches to rescue CFTR function.

## Figures and Tables

**Table 1:** List of rare (non-F508del) genotypes used in this study, their FDA approval status for Trikafta or Kalydeco, and their FIS values for ETI (FIS_ETI_), ivacaftor (FIS_IVA_), or basal FIS in the absence of modulators (FIS). FDA approval is indicated as YES/NO for each allele (approval of clinical eligibility of a single allele is considered sufficient for the genotype). FIS values are relative to the values of the corresponding non-swelling organoids.

Sample	Genotype	FDA+ TRIKAFTA Allele1/Allele2	FIS_ETI_ Mean (SD)	FDA+ KALYDECO Allele1/Allele2	FIS_IVA_ Mean (SD)	FIS Mean (SD)
**CF Patients with Rare Genotypes**						
CF25	R1066C/G542X	NO/NO	1.60 (0.15)	NO/NO	1.01 (0.02)	1.03 (0.03)
CF26	N1303K/R764X	YES/NO	1.16 (0.04)	NO/NO	1.07 (0.03)	1.15 (0.05)
CF27	N1303K/N1303K	YES/YES	1.28 (0.07)	NO/NO	1.04 (0.04)	0.98 (0.02)
CF28	G85E/I1234V	YES/NO	1.54 (0.16)	NO/NO	1.29 (0.10)	1.25 (0.06)
CF29	N1303K/N1303K	YES/YES	1.22 (0.08)	NO/NO	1.07 (0.06)	1.12 (0.02)
CF30	G85E/G85E	YES/YES	1.79 (0.23)	NO/NO	1.19 (0.12)	1.09 (0.07)
CF31	L1077P/L1077P	YES/YES	1.91 (0.11)	NO/NO	1.04 (0.03)	1.06 (0.07)
CF36	G85E/2183AA>G	YES/NO	1.62 (0.09)	NO/NO	1.00 (0.03)	0.98 (0.08)
CF37	N1303K/R1066H	YES/YES	1.98 (0.22)	NO/NO	1.15 (0.04)	1.06 (0.01)
CF39	L1077P/W1282X	YES/NO	2.11 (0.15)	NO/NO	1.09 (0.05)	1.07 (0.14)
CF49	3849+10kbC>T/dele22-24	YES/NO	1.74 (0.12)	YES/NO	1.69 (0.22)	1.14 (0.05)
CF50	W1282X/W1282X	NO/NO	1.02 (0.02)	NO/NO	1.06 (0.03)	1.09 (0.03)
CF52	S549R/G542X	YES/NO	1.95 (0.16)	YES/NO	1.29 (0.15)	1.20 (0.04)
CF55	L1077P/R1066C	YES/NO	1.94 (0.16)	NO/NO	1.00 (0.01)	1.00 (0.05)
CF56	TG13T5/[L24F;296+2T>G]	YES/NO	1.82 (0.10)	NO/NO	1.85 (0.12)	1.44 (0.03)
CF57	N1303K/R334W	YES/NO	1.66 (0.21)	NO/NO	1.60 (0.01)	1.12 (0.05)
CF59	N1303K/H139R	YES/YES	1.70 (0.14)	NO/NO	1.07 (0.08)	1.04 (0.03)
CF61	Q1291R/W1282X	YES/NO	2.43 (0.13)	YES/NO	2.01 (0.14)	1.22 (0.06)
CF62	Q1291R/W1282X	YES/NO	2.74 (0.10)	YES/NO	2.03 (0.11)	1.25 (0.05)
CF64	Q1291R/W1282X	YES/NO	2.61 (0.15)	YES/NO	2.16 (0.22)	1.12 (0.04)
CF65	N1303K/I444T	YES/NO	1.87 (0.09)	NO/NO	1.08 (0.05)	1.01 (0.06)
CF66	N1303K/I444T	YES/NO	1.97 (0.13)	NO/NO	1.17 (0.04)	1.03 (0.08)
CF67	R334L/G542X	YES/NO	2.16 (0.05)	NO/NO	1.38 (0.08)	1.10 (0.13)
CF70	[R117L;L997F]/R334W	NO/NO	1.86 (0.10)	NO/NO	1.46 (0.10)	1.23 (0.08)
CF71	[R117L;L997F]/R334W	NO/NO	1.89 (0.23)	NO/NO	1.70 (0.18)	1.15 (0.04)
CF72	N1303K/3849+10kbC>T	YES/YES	2.15 (0.11)	NO/YES	1.28 (0.03)	1.18 (0.05)
CF73	N1303K/N1303K	YES/YES	1.48 (0.05)	NO/NO	1.03 (0.07)	1.06 (0.06)
CF74	H139R/H139R	YES/YES	2.05 (0.17)	NO/NO	1.14 (0.08)	1.14 (0.04)
CF75	D110H/[V562I;A1006E;TG11T5]	YES/NO	2.29 (0.10)	YES/NO	1.82 (0.09)	1.56 (0.16)
CF76	N1303K/D614G	YES/YES	2.57 (0.08)	NO/NO	1.73 (0.17)	1.74 (0.14)
CF77	R334W/711+1G>T	NO/NO	1.66 (0.15)	NO/NO	1.20 (0.10)	1.22 (0.15)
CF80	D614G/R334L	YES/YES	2.08 (0.23)	NO/NO	1.89 (0.21)	1.41 (0.06)
CF81	711+3A>G/R334L	YES/YES	1.51 (0.01)	YES/NO	1.53 (0.09)	1.15 (0.06)
CF82	711+3A>G/R334L	YES/YES	1.78 (0.17)	YES/NO	1.46 (0.09)	1.26 (0.07)
CF97	G542X/G542X	NO/NO	1.05 (0.06)	NO/NO	1.06 (0.07)	1.08 (0.03)
CF98	2183AA>G/c.1584+18672A>G	NO/NO	1.26 (0.04)	NO/NO	1.16 (0.02)	1.08 (0.06)
CF101	621+1G>T/dup ex6b-ex16	NO/NO	1.01 (0.05)	NO/NO	1.07 (0.02	1.06 (0.07)
CF105	G551D/621+1G>T	YES/NO	2.38 (0.24)	YES/NO	1.36 (0.11)	1.00 (0.03)

## Data Availability

The original contributions presented in this study are included in the article/Supplementary Materials. Further inquiries can be directed to the corresponding author.

## Supplementary Materials

The following supporting information can be downloaded at https://www.mdpi.com/article/10.3390/cells14231914/s1. Table S1: Patient’s clinical information; Table S2: Nasal organoids with reference genotypes used for FIS assay and their FIS response; Figure S1: Characterization of nasal organoids via confocal laser scanning microscopy (CLSM) and immunoblot analysis; Figure S2: Forskolin-induced swelling (FIS) assay of nasal organoids with reference genotypes; Video S1: Differentiated nasal organoid showing beating cilia oriented towards the lumen.
