# Supplementary material for: CFTR Modulator Response in Nasal Organoids Derived from People with Cystic Fibrosis
Source: Cells. 2025 Dec 2;14(23):1914. doi: 10.3390/cells14231914 (PMC12691081; doi:10.3390/cells14231914)
Supplement: Supplementary file 1 [file cells-14-01914-s001.zip › cells-3937446-supplementary.pdf]

Supplementary information

Code	Genotype	Average sweat test value (mEq/l)	FEV$_1$ %	Pancreatic insufficiency (Y/N)	Lung disease severity (exacerbations/year)
CF25	R1066C/G542X	80	39%	Yes	7 severe
CF26	N1303K/R764X	97	67%	Yes	4 severe
CF27	N1303K/N1303K	86	55%	Yes	4 severe, 4 mild
CF28	G85E/I1234V	71	44%	Yes	3 severe
CF29	N1303K/N1303K	72	80%	Yes	2 severe, 3 mild
CF30	G85E/G85E	86	29%	Yes	lung transplanted
CF31	L1077P/L1077P	97	44%	Yes	3 severe, 3 mild
CF36	G85E/2183AA>G	94	63%	Yes	5 severe
CF37	N1303K/R1066H	96	35%	Yes	5 severe 2 mild
CF39	L1077P/W1282X	93	39%	Yes	3 severe 5 mild
CF49	3849+10KbC>T/dele22-24	61	80%	No	3 mild
CF50	W1282X/W1282X	77	25%	Yes	5 severe, 2 mild
CF52	G542X/S549R	85	65%	Yes	3 mild
CF55	L1077P/R1066C	97	87%	Yes	2 mild
CF56	TG13T5/L24F;296+2T>G	76	101%	No	1 mild
CF57	R334W/N1303K	79	57%	No	1 severe, 3 mild
CF59	N1303K/H139R	69	86%	Yes	3 mild
CF61	W1282X/Q1291R	57	46%	No	2 severe, 2 mild
CF62	W1282X/Q1291R	60	65%	No	1 severe, 1 mild
CF64	W1282X/Q1291R	61	28%	No	3 severe, lung cancer
CF65	N1303K/I444T	72	100%	No	no
CF66	N1303K/I444T	76	97%	No	no
CF67	G542X/R334L	72	101%	No	no
CF70	R117L;L997F/R334W	71	70%	no	no
CF71	R117L;L997F/R334W	75	118%	No	no
CF72	N1303K/3849+10kbC>T	57	125%	No	no
CF73	N1303K/N1303K	82	62%	Yes	3 severe, 4 mild
CF74	H139R/H139R	90	120%	No	no
CF75	D110H/V562I;A1006E;TG11T5	49	106%	No	no
CF76	N1303K/D614G	60	110%	No	no
CF77	R334W/711+1G>T	94	96%	Yes	1 mild
CF80	D614G/R334L	65	103%	No	no
CF81	711+3A>G/R334L	33	107%	No	no
CF82	711+3A>G/R334L	48	72%	No	no
CF97	G542X/G542X	96	45%	yes	3 severe
CF98	2193AA>G/c.1584+18672A>G	84	38%	no	4 severe
CF101	621+1G>T/dup ex6b-ex16	84	27%	Yes	4 severe, 4 mild
CF105	G551D/621+1G>T	85	34%	Yes	3 severe, 4 mild
CF13	F508del/F508del	87	52%	Yes	3 mild
CF18	F508del/F508del	93	30%	Yes	1 mild
CF43	F508del/F508del	99	71%	Yes	no
CF54	F508del/F508del	75	81%	Yes	no
CF10	F508del/F508del	85	38%	Yes	2 mild
CF53	F508del/F508del	74	36%	Yes	3 mild
CF42	F508del/R553X	97	73%	Yes	2 mild
CF46	F508del/W1282X	80	24%	Yes	1 severe, 2 mild
CF51	F508del/E585X	68	87%	Yes	1 severe, 2 mild

Table S1. Patient clinical information. Sample code, genotype, sweat test values, FEV1 %, pancreatic sufficiency of insufficiency and lung disease severity (exacerbations/year) are indicated for each of the 38 patients that participated in this study.

Clinical data refer to untreated patients, except those related to genotypes bearing F508del variant (CF13 to CF51) or to cases CF52 and CF105, whose clinical data refer to patients under treatment (ETI or ivacaftor, respectively).

SAMPLE		GENOTYPE	FIS mean (SD)	mean (SD)	FIS_{IVA} mean (SD)	mean (SD)	FIS_{ETI} mean (SD)	mean (SD)
**WILD TYPE**	CF24	N/N	2.33 (0.04)	2.32 (0.26)				
	CF41	N/N	2.57 (0.06)					
	CF69	N/N	2.05 (0.08)					
**CARRIER**	CF4	[G576A;R668C]/N	1.96 (0.15)	2.22 (0.21)				
	CF103	dup15-22/N	2.25 (0.05)					
	CF104	[1210-34TG10_1210-34TG4ins317]/N	2.42 (0.17)					
**F508del/F508del**	CF10	F508del/F508del	1.01 (0.02)	1.05 (0.05)	1.01 (0.04)	1.04 (0.03)	1.86 (0.11)	1.94 (0.19)
	CF13	F508del/F508del	1.01 (0.06)		1.02 (0.03)		2.29 (0.03)	
	CF18	F508del/F508del	1.06 (0.05)		1.01 (0.01)		1.93 (0.08)	
	CF43	F508del/F508del	1.06 (0,05)		1.05 (0.11)		1.77 (0.02)	
	CF53	F508del/F508del	1.03 (0.05)		1.04 (0.05)		1.96 (0.24)	
	CF54	F508del/F508del	1.15 (0.06)		1.10 (0.08)		1.81 (0.05)	
**F508del/null**	CF42	F508del/R553X	1.09 (0.12)	1.02 (0.07)	1.04 (0.02)	1.04 (0.06)	1.65 (0.06)	1.66 (0.15)
	CF46	F508del/W1282X	1.02 (0.02)		0.99 (0.08)		1.51 (0.16)	
	CF51	F508del/E585X	0.96 (0.02)		1.10 (0.15)		1.82 (0.15)	
**null/null**	CF50	W1282X/W1282X	1.09 (0.03)	1.07 (0.02)	1.06 (0.03)	1.07 (0.05)	1.02 (0.02)	1.02 (0.02)

Table S2. Nasal organoids with reference genotypes used for FIS assay and their FIS response. FIS assay values (FIS, FIS_{IVA} and FIS_{ETI}) of the nasal organoids of each sample and mean for each group (wt, carrier, F508del/F508del, F508del/null, null/null) of genotypes (null is a CFTR variant with no residual function and unresponsive to modulators, specifically a nonsense mutation variant). Organoids with wt-CFTR or carriers were treated with forskolin to determine the extent of functional CFTR activity (wt allele in double or single copies) in the FIS assay. The mean FIS value is expressed as the ratio of organoids mean area after forskolin treatment versus the same organoids before treatment (normalized to non-treated organoids).

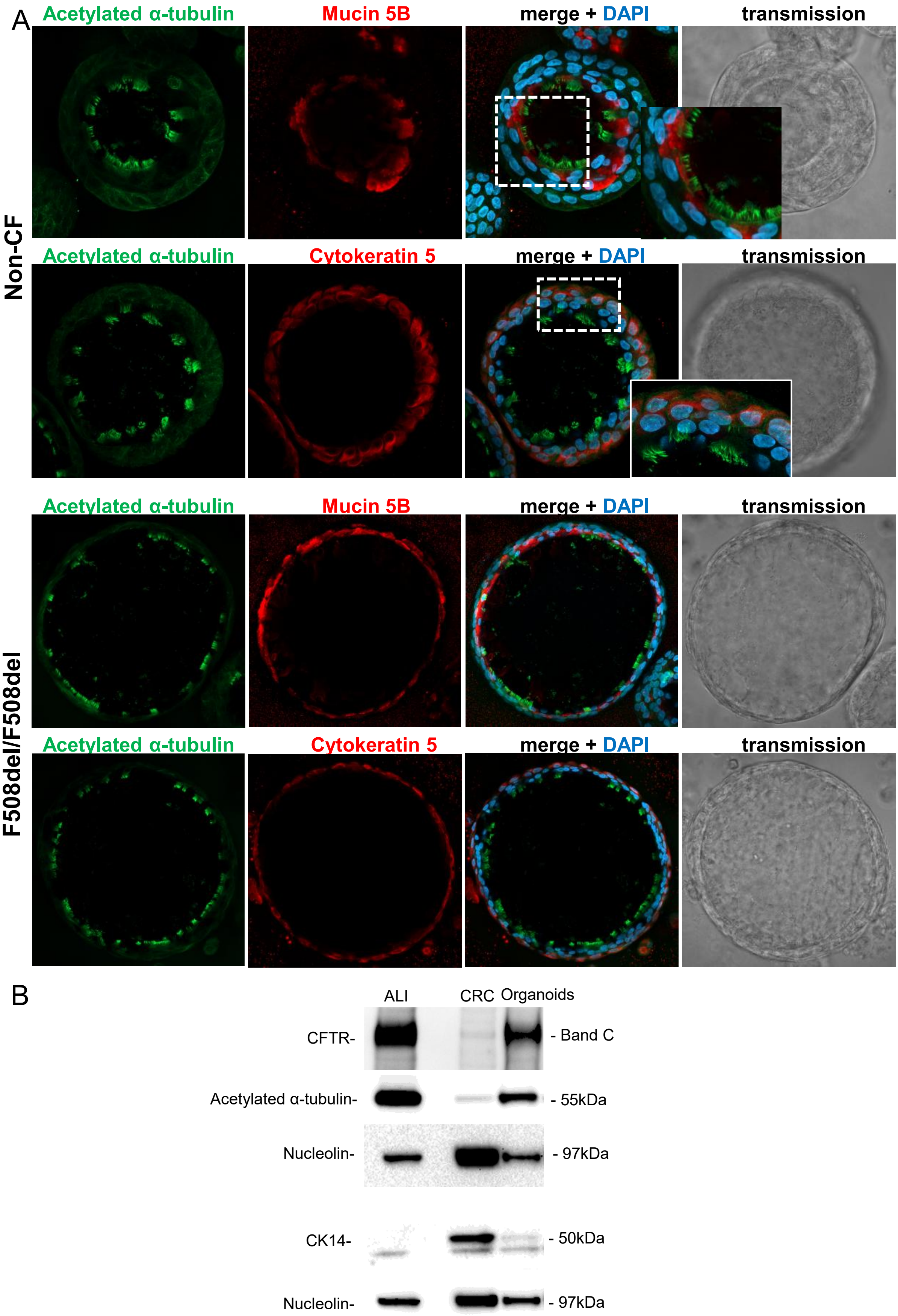

Figure S1. Characterization of nasal organoids by confocal laser scanning microscopy (CLSM) and immunoblot analysis. A) For CLSM analysis organoids were fixed, permeabilized and stained with anti-acetylated α-tubulin (green) and anti-Mucin 5B (red) or anti-acetylated α-tubulin (green) and anti-cytokeratin 5 (red). Nuclei are stained in blue (DAPI). Separate channels and merged images are shown. Insets represent higher magnification images of the selected region of interest. A transmission light image is reported (right) with arrows indicating cilia in the luminal side of the

organoids. Scale bars, 20 µm. Images from one representative experiment out of three non-CF samples and one out of 6 CF samples (F508del/F508del) performed are shown, as indicated. B) Representative images of immunoblot analysis of CFTR, acetylated alpha tubulin, cytokeratin 14 (ck14) and nucleolin (for equal loading expression) in non-CF CRC cells and in CRC-derived organoids as indicated. Positive control sample (ALI) was loaded for reference (CRC-derived ALI-cultures obtained as previously reported [23-25].

In vitro expanded cells were banked (10 vials of CRC cells were stored for each patient sample, yielding, as an approximate estimation, a total of > 5 × 10^8 CRC, starting from a mean of $5x10^4$ nasal brushing cells). Routine validation for stem cell- and epithelial-marker expression (to confirm the airway basal cell nature) and CFTR gene sequence analysis (to confirm the presence of the pathogenic variants previously detected during diagnostic procedures) were performed for each sample, following our previously published protocols [23-25, 32]. Diagnostic pathogenic variants were confirmed in patient CRC cell-derived DNA after in vitro expansion, demonstrating that CRC cells maintain the patient's genotype after prolonged culture, in agreement with our previous data, thus constituting a suitable disease model for the investigation of CFTR variants in a personalized manner. CRC cells were used to generate organoids (as described in the Materials and Methods section) whose full differentiation was demonstrated by proving respiratory cell marker expression and observation of cilia movement under optical microscopy in the luminal side of the organoid wall. Phenotypic, functional and genetic validation of organoids was performed for all samples. In the figure a representative phenotypic validation of organoids. Organoids replicated the differentiation status of the respiratory tissue of origin, with the formation of a polarized organoid wall constituted by mucin-producing cells dispersed within the organoid wall, residual undifferentiated cytokeratin 5 $(ck5)^+$ basal cells localized at the external side of the wall and ciliated cells with beating cilia oriented toward the lumen (Supplementary Figure 1A and supplementary Movie 1. CFTR protein was expressed at low levels in CRC cells and induced following differentiation under organoid culture conditions, in parallel with increased expression of acetylated alpha tubulin and decreased expression of the basal stem cell marker cytokeratin 14 (ck14), as occurred and previously shown for ALI-cultures (Supplementary Figure 1B) [23-25].

Supplementary
Movie 1.mp4

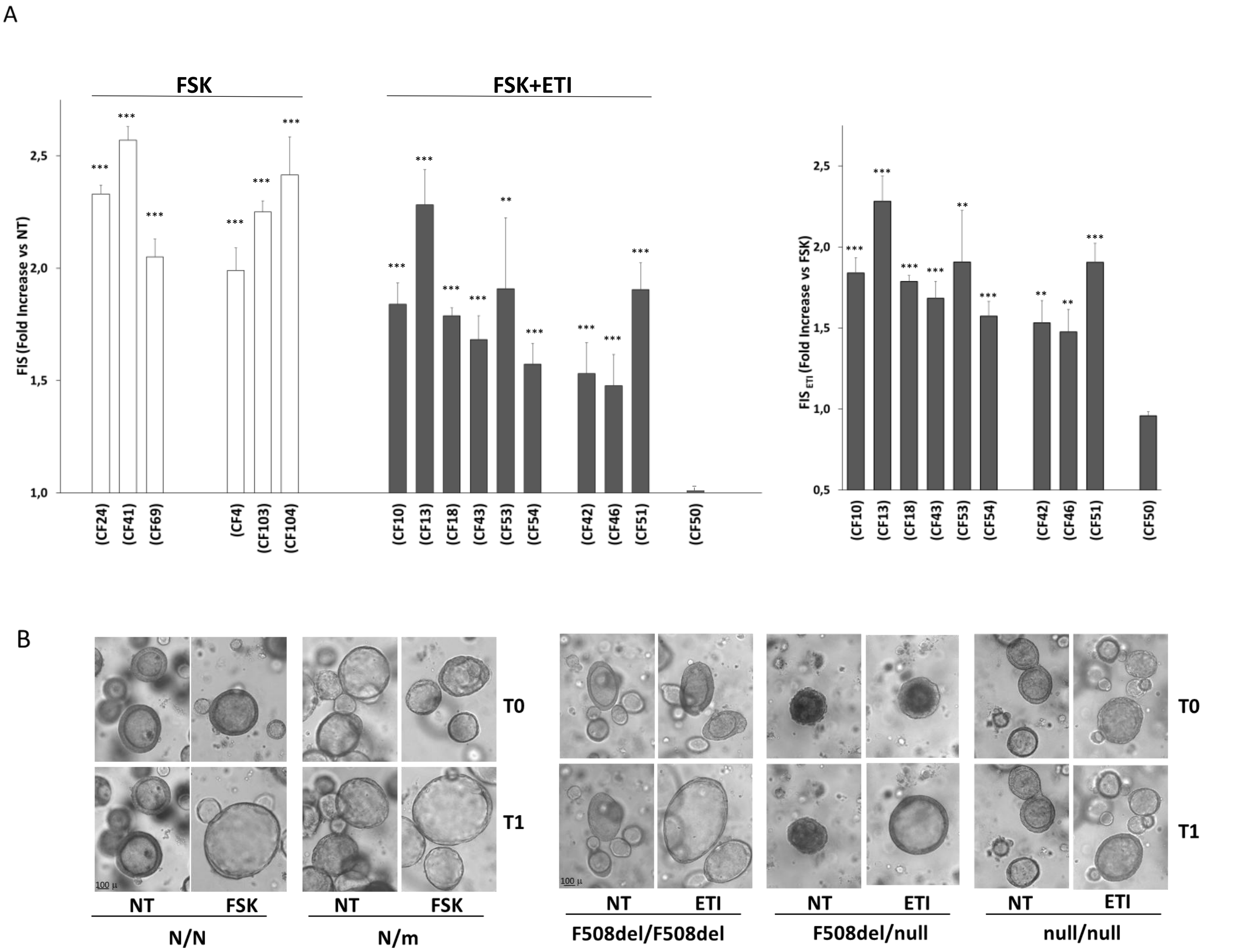

Figure S2. Forskolin-induced swelling (FIS) assay of nasal organoids with reference genotypes. A) The histograms show nasal organoid FIS results on the following 4 sets of control genotypes: wt (N/N), carrier (N/m), F508del/F508del, F508del/null. FIS values are relative to the values of the corresponding non-swelling (left) or fsk-treated organoids (right). Treatment with modulators (Tezacaftor, Elexacaftor, Ivacaftor=ETI) was performed for CF samples, while wt or carrier genotypes were treated with fsk-only, as indicated.

B) Representative images of FIS assay organoids are reported relative to the indicated genotypes. The same organoids, monitored and measured before (T0) and after 48 hours of fsk or fsk+modulators (T1) treatments, are shown. Measures are relative to the increase in the organoid outer area (mean of at least 30 organoids per condition) after stimulation with the indicated drugs. Statistical significance of difference with respect to the untreated control value was analyzed using the ANOVA test: **$p < 0.01$; ***$p < 0.001$. Scale bars (100 µm) are indicated on the lower-left organoid image for each genotype and are representative of all organoid images.
